# Reconsidering Autonomy: Asian Americans’ Use of Relational Autonomy in Organ Donation Decisions

**DOI:** 10.21203/rs.3.rs-5110495/v1

**Published:** 2024-10-30

**Authors:** Gerard P. Alolod, Diana C. Litsas, Laura A. Siminoff

**Affiliations:** Temple University; Temple University; Temple University

**Keywords:** Autonomy, relational autonomy, Think Aloud, end-of-life, organ donation, organ procurement organization

## Abstract

**Background:**

As cultural contexts have gained increasing relevance in medical decision-making, the current mainstream definition of autonomy is insufficient. A viable alternative framework, relational autonomy posits that agents’ actions are influenced by and embedded in society and culture rather than occurring in isolation. To test the concept’s applicability, we examine whether Asian Americans in the study’s sample operationalize relational autonomy as a decisional approach in hypothetical scenarios about organ donation, a practice for which there is considerably lower enthusiasm compared to other racial groups in the US.

**Methods:**

A national sample of Asian American adults were recruited from Qualtrics research panel. Participants completed a Think-Aloud interview containing scenarios in which they decide whether or not to: 1) become a registered donor at the motor vehicle department; 2) authorize organ donation for a close relative who unexpectedly died. The interview first elicited candid reactions to the scenarios, followed by probing participants’ rationale of their initial responses. Participants’ final decision to each scenario (whether or not to register; whether or not consent to surrogate authorization), as well as participants’ decisional approaches (individualistic vs relational) were coded using the constant comparison method.

**Results:**

The sample (n = 40) mirrored the largest proportions of Asian Americans in the US; the plurality identified as Chinese (35%), Filipino (27.5%) and Indian (25%). In response to the organ donor registration prompt, a majority of respondents (57.5%) expressed they would employ the mainstream decisional approach of individualistic autonomy, and 42.5% would make the decision with a relational approach. In contrast, when responding to the surrogate authorization prompt, the majority (77.5%) described a relational approach when making the decision, to preserve familial harmony and honor their cultural heritage.

**Conclusions:**

Use of individualistic and relational autonomy frameworks are situational for some individuals. Participants acknowledged the impact of personal, cultural, and societal elements on their decisional approach. The concept of relational autonomy has utility through its versatility in complex decision-making events and by accounting for multiple stakeholders without privileging the autonomy of a single decision-maker over others.

## BACKGROUND

Ethical and informed decision-making is an essential component of quality healthcare, especially in the context of sensitive health topics, such as end-of-life and post-mortem organ donation. While a cornerstone of decision-making is respect for autonomy, understanding the cultural context of autonomy is also an important factor and is spurring a reconsideration of the meaning and implementation of decisional autonomy (1–7). Beauchamp and Childress’ classic definition of autonomy derives lineally from Kant and is defined as a “form of personal liberty of action where the individual determines his own course of action in accordance with a plan chosen by him- or herself. The autonomous person is one who not only deliberates about, and chooses such plans, but can act based on such deliberations” (8). As such, the ideal of autonomy is that it resides wholly within an individual and is exercised unilaterally. This formulation of autonomy is now largely codified in laws, policies used by Institutional Review Boards governing informed consent for research, and in contemporary Western biomedical practice (9–12).

More recently, however, theorists and researchers have argued that this commonly accepted understanding of autonomy fails to address the increasing complexities of medical practice, is intrinsically patriarchical (13), and is devoid of cultural context (14–17). This scrutiny reveals the inadequacy of the current mainstream definition of autonomy, which is highly prescriptive, derives from a single cultural and philosophical tradition, and does not capture the contextual and complex nature of medical decision-making. Bishop has criticized the highly individualized nature of informed consent as a ”myth,” arguing that individuals never really make decisions in complete isolation (18). In addition, recent literature has highlighted the tensions between the Western implementation of informed consent and individualistic autonomy and different Asian contexts (19,20), in which individual patients are inextricably situated within family units. Thus, scholars have increasingly deemed the individual-directed model of autonomy as essentially incompatible with understudied cultural settings and have called for its revision (17,21,22).

### Relational Autonomy as an Alternative Framework

Proposed as a viable, alternative framework, relational autonomy has foundations in feminist ethics and is an umbrella term for views on autonomy that agents do not act in isolation and without influence of society and culture (13). Building on the work of Sherwin and colleagues (23,24), the current study operationalizes relational autonomy as a decisional approach of autonomy wherein decisions are made with consideration of and in conjunction with one’s relationships and within particular social, political, and economic conditions. Thus, the individual decision maker still exercises autonomy, but decision-making is derived from his/her embeddedness in family and society.

Relational autonomy has support in empirical research and has become a useful lens for scientific inquiries about end-of-life decision-making among Asian and Asian American populations by accounting for values like the primacy of the family unit, holistic harmony, and familial duty (1,25–27). Extant work has suggested a movement away from individualistic notions of the patient as the sole decision-maker by demonstrating the critical roles played by a patient’s family (28–32). Other research has indicated that some patients even prefer to cede medical decision-making authority to family members (33,34).

The topic of organ donation authorization offers insight to the range and complexity of possible decision-making approaches. There is a need to understand whether and how relational autonomy might be exercised among Asian Americans, whose own transplant needs are disproportionately high (35) due to higher rates of hepatitis and liver cancer, for which transplantation is the only curative option (36). Indeed, the proportion of Asian Americans on the US transplantation waitlist is nearly 40% higher than that of the general population (37,38). Nevertheless, Asian Americans have amongst the lowest donation rates in the US (39), resulting in lower odds of finding a donor match (40). This public health challenge is especially urgent given that Asian Americans are the fastest growing racial group in the country (35).

The available research about Asian Americans and organ donation has largely focused on attitudinal and knowledge barriers toward organ donation (25,41–47).

### Objective

By focusing on how Asian Americans approach the decision to (1) become a registered organ donor and (2) donate the organs of a deceased family member, the current study examines whether decisions related to organ donation are situated within perceived family views and the needs of their communities. This study sought to explicitly explore how Asian Americans make decisions for organ donation by examining how individual and relational autonomy are exercised in making these decisions. We also test whether decision-making approaches are situational or stable across two classic organ donation scenarios.

## METHODS

This study builds on and was part of a larger investigation to identify the assets and barriers to organ donation among Asian American populations regionally and nationally. Guided by local stakeholder organizations representing Asian American communities and a community advisory board (CAB), earlier phases involved focus groups (25), the development and deployment of the largest national survey conducted on Asian Americans’ donation-related attitudes, behaviors, and knowledge to date (41), and the creation and testing of a social media campaign to increase awareness and encourage donor registration (42),

### Sample and Recruitment

The research team utilized research panel services from Qualtrics LLC to facilitate, identify, and recruit a pool of panelists. Researchers sent initial study invitations by e-mail and conducted follow-up recruitment phone calls using a screening tool to confirm that participants self-identified as Asian American, were ≥ 18 years of age and willing to participate in a telephone interview. Effort was made to generate a pool of participants that proportionately approximated the Asian American population. Individuals who worked in healthcare were excluded from the study. The study was deemed exempt by the Temple University IRB (#25254).

### Data Collection and Measurement

Study participants completed a self-administered survey and a 60-minute interview. The survey collected social and demographic data, including gender, ethnicity, age, marital status, education level, total household income, country of birth, and year of immigration to the US, if applicable. With demonstrated validity in past studies (48–51), the interview employed a ‘Think Aloud’ method to elicit candid and spontaneous responses from study participants about medical decision-making (52). An interview guide was developed and consisted of two sections. In Section 1, participants were presented with two organ donation-related scenarios: 1) becoming a registered organ donor at the motor vehicle department, and 2) deciding about surrogate donation, or the donation of a family member’s organs in hospital following an unexpected death. (See Table 1) In Section 2 of the Think Aloud Interview, respondents were asked to describe the rationale behind their medical decision-making for each scenario. This provided participants the opportunity to verify their initial choices, clarify, and further elaborate for a fuller understanding of their responses.

The main outcome of interest across both scenarios was the approach to decision-making, defined as individualistic or relational. Individualistic decision-making, based in the current normative definition of autonomy, considers only the wishes of the respondent. For example, a decisional approach was deemed individualistic if participants’ responses emphasized that the decision was theirs alone to make. Relational decision-making was defined as considering cultural norms and societal obligations, including consultation with or seeking to respect the wishes of family members.

Interviews were conducted by research staff trained in qualitative methods and were conducted via telephone or Microsoft Teams (Microsoft, Redmond, WA), depending on the participant’s preference. Interviews were audio-recorded and stored on a HIPAA-compliant platform. Participants received a $100 gift card after completing the interview. Recordings were de-identified and transcribed in preparation for analysis.

Interview transcripts were uploaded to MAXQDA 2022 (VERBI Software, 2021). The domains from the interview guide were used as major coding categories for an initial coding schema, and additional codes were developed inductively using the constant comparative method (53). Codes were assigned for decisions about donor pre-designation (would register/would not register) and surrogate donation (authorize/refuse/unsure). Additionally, utterances were coded to denote the decisional approach of each respondent, either individualistic or relational. Two qualitatively trained research staff coded the transcripts, and a cultural anthropologist (GPA) oversaw this process to avoid coding drift. To ensure reliability, ten percent of the transcripts were selected at random and analyzed by an independent coder (GPA); an overall interrater reliability of 91.2% agreement was achieved.

Descriptive statistics and frequency counts were calculated for reported sociodemographic data, scenario outcomes, and decisional approach. Sub-group analyses were also conducted by scenario outcome and decisional approach; a nonparametric measure of association (i.e. Fisher’s exact) measured the association between these and sociodemographic characteristics, such as sex, ethnicity, being born or raised in the US (yes/no), educational attainment, age group (< 55 and ≥ 55 years), or household income level.

## RESULTS

### Sample Characteristics

Services provided by Qualtrics (described above) resulted in a pool of 116 potential participants from which 40 participants were contacted and completed an interview between November 2021 and January 2022. A plurality of participants self-reported ethnicity as Chinese (35%), Filipino (27.5%) and Indian (25%); 50% were female (see Table 2). The median age was 51.5 years with over half (52.5%) under 55 years, mirroring the median age of the decision-making population nationally.(54) More than half the sample were born or raised outside of the US (55%) and reported a median age of 24 years at time of immigration. Most participants were married or cohabitating (62.5%) with 92.5% having attained formal education beyond a high school degree and 45% reporting an annual household income of over $80,000. Interviews ranged from 29 to 94 minutes with a mean duration of 49 minutes.

### Registration and Organ Donation Decisions

Study participants were presented with the scenario of being asked to register as an organ donor while renewing a driver’s license (See Table 1 for scenarios.) A majority of respondents (65.0%; n=26) expressed that they would be willing to register as an organ donor (Table 3). A second scenario (Table 1) described a decision to donate a family member’s organs post-mortem. Half of participants (50.0%; n=20) would authorize donation whereas 32.5% (n=13) would refuse donation, and 17.5% (n=7) were undecided (Table 4). Fisher’s exact test examined whether a decisional approach (i.e. individualistic vs. relational) was associated with decisions to register as a donor (Scenario 1) or donate a family member’s organs on death (Scenario 2). We found no statistically significant associations between decisional approach and choice outcomes with sex, ethnicity, being born or raised in the US (yes/no), educational attainment, age group (<55 and ≥55), or household income level.

### Decisional Approach in Becoming a Registered Organ Donor

#### Individualistic Approach to Autonomy

We examined how individuals made decisions about whether to become an organ donor by using the ‘Think Aloud’ methodology to help participants explain *how* they made their decisions. Of the 26 participants who would choose to register as a donor, 53.8% (n=14) approached decision-making individualistically. These participants expressed a belief that the decision was purely personal in nature and that there was no need for others’ input. Participants also explained their individualistic approach toward donor registration as not requiring approval from or consultation with others. Even for a participant, who had previously spoken about the importance of his family’s advice, stated that he would not want anyone else’s input about registering as an organ donor. He replied, “No… I’ll make the decision. I would not ask anyone for advice to whatever” (ID 112746; 45-year-old Chinese American). Recalling her last license renewal, a 67-year-old Chinese American female participant expressed that she was able to make the determination on her own and did not want others to interfere. She stated

… it is a very personal decision, and I don’t need any other help in making that decision. I don’t really want to be swayed either way and this is something that you have to be comfortable yourself instead of “person A said this, and person B said something else.” So, no I did not. Mainly my own decision. And it is only your own decision that you can stand by it. (ID 48240)

Similarly, an 18-year-old Korean American female asserted “I don’t think I’m going to consult with anyone because that’s my personal choice. And if I wanted to donate my organs, I don’t think anybody else has a say…” (ID 74511).

#### Relational Approach to Autonomy

A sizable proportion of the sample (42.5%; n=17) used a relational decisional framework to decide whether or not to register as an organ donor. For this set of participants, the decision was less about personal choice and more about factors of family expectations and culture or religious norms. Although donor registration at the motor vehicle’s department is typically completed independently, these participants stated that they would factor in the opinions and input of their families. For example, when asked if she would consult anyone about the decision, a 23-year-old Filipina American said, “I would refer to my family members [with] the final decision…I know that being an organ donor would save lives, but my family members’ [perspective] has much more priority than mine” (ID 83501). Similarly, another member of the sample responded, “I think I will talk to my husband at first… He’s the one that always helps me to figure things out.” (ID 98664; 35-year-old Indian American female)

Other participants based their decision because they personally experienced the need for donation within their families and were acutely aware of the societal need for organs. A 33-year-old Pakistani American male participant explained

[The decision] came naturally to me because some of my family members have had kidney transplants and stuff. So I know that there is a need for organs and everything. So I didn’t even think about it. I just like checked it. (ID 32026)

A 63-year-old Indian American male likewise shared, “I had some relatives who had kidney transplant[s]. They were the recipient. So, I know the importance of it” (ID 19208).

Other interviewees referred specifically to cultural conventions in their decision-making rationale. One participant affirmed

I know [being a registered organ donor] is a good thing to do, but I just haven’t gotten to the point where I’m willing to donate my organs. And I don’t know why. That might be a cultural thing…I think in the Asian culture, when you die, you’re buried whole. And I think that’s instilled in me. So even though I know it’s better to donate, but I haven’t brought myself or mentally prepared to do that. (ID 77138; 63-year-old Chinese American female).

Religion was also specifically cited to justify whether or not to become a registered organ donor. One participant said,

[I]n our religion [Jain], it’s a cremation. We don’t bury. So, this dead body is going to be cremated. So, whether there is a heart or kidney inside is not different. But see if you can help someone prolong their life, that’s good. Someone give you blessing. Someone will appreciate it. And maybe then they can pay it forward, or their family can pay it forward. (ID 19208; 63-year-old Indian American male)

### Decisional Approach in Surrogate Organ Donation

#### Individualistic Approach to Autonomy

Participants were asked to respond to a second scenario in which they were asked to consider donating the organs of a close family member on death (Table 1). A minority (22.5%; n=9) of the sample made their decision using an individualistic approach. This small segment of participants articulated their sole authority as a surrogate decision maker. For example, while considering the donation of his parents’ organs, a 32-year-old Korean male asserted

I’d have to use all the circumstances around me to make the best decision, which means ultimately that I’m coming up with the decision on my own. If I think that [my parents’] organs could help people here, that supersedes their own traditions. (ID 87740)

Other participants reported that they would act based on the donor’s wishes to the best of their knowledge. For instance, one interviewee referred to the absence of donor registration. She explained:

Well, since he didn’t sign up for anything, it is basically my family member’s decision, and it is not me... I would not do anything about it because it’s just you respecting the person… So I am not doing anything about it. (ID 22105; 40-year-old Filipina American)

A 19-year-old Chinese American female participant said similarly

I wouldn’t donate the organs... especially if they didn’t indicate before death what they wanted to do with their organs. So, I think it’s a pretty cut and dry situation for me. I wouldn’t do it. (ID 36111)

Another interviewee responded similarly, saying “I mean it’s a very tricky question. But no, I will not donate organs. Unless I’ve discussed it with that family member and I know his or her wishes, I won’t” (ID 23757; 41-year-old Indian American male). Notably, the participants cited above all similarly used an individualistic decisional rationale but arrived at different decisions about surrogate donation.

#### Relational Approach to Autonomy

In contradistinction, a large majority (77.5%; n=31) described their decisional process in terms of what we defined as relational autonomy. This approach was used to avoid family conflict and to honor their cultural heritage. Participants were cognizant of the lifesaving aspects of organ donation and wanted to donate but only if it did not disrupt the harmony of the family. For example, a 23-year-old Filipina American participant who was personally in favor of organ donation stated:

[I]f I have been in an accident like that… there’s no recovery for me. And then I think my parents or my family members will disagree about donating my organs, cause at first, being in an accident will be a terrorizing thing… I think for me, at the end, I’m not decided about this, but I think I would refer to my family members, the final decision, as I know that being an organ donor would save lives, but my family members have much more priority… (ID 83501)

A On 43-year-old Chinese American female participant also indicated the need for conferring with family members to avoid conflict:

If it’s my parents, I think I would pause and consult the other parent. So, if it’s my mom, I think I would consult my dad—at least ask and tell him my preference and ask. If it’s my son, I will consult with my husband. So, I do think there is some contingent relationship. I don’t want them to be surprised, and I will tell them my leanings and just make sure they don’t disagree. But, if they disagree, I will say ‘no’ [to organ donation] because I don’t think it’s something that I feel so strongly enough that I want to fight over it. I’m hoping they will say yes. (ID 71200)

Another participant described needing to obtain his family’s consent:

I have to take the consent of other family members and maybe convince them. It would not be my decision. Like it would be a consensus decision by family members with consultation and like other stuff. There might have been some heated discussions and stuff depending on the situation and the nature of relationship [to the deceased]… So, I mean, there’s this angle of mutilating the body of a dead person or something like that. Like some family members might have that, thinking that it’s kind of disrespectful to remove organs and stuff. So, you have to navigate very carefully. (ID 32026, 33-year-old Pakistani American male)

An 18-year-old Korean female interviewee indicated that she would defer to her brother in the scenario. She explained:

Just the fact that I might be naïve or innocent or emotional, so I might not think straight in the moment. He’s very logistical… He’s like, “This is what mom and dad would have wanted and this is what we’re gonna do.” (ID 74511)

Those who adopted a relational decisional framework also cited individuals in need of organ donation. A 32-year-old Indian American female participant said, “Like [my family member] couldn’t survive, but maybe they can save someone else’s life by their organs being donated” (ID 29392). Another interviewee stated, “Go ahead! Go ahead and take whatever you need... After all, a life is lost on one hand. Why not give somebody else a better chance of a better life on the other?” (ID 68534, 65-year-old Chinese American female). Similarly, one respondent recalled learning about the impact of organ donation saying, “sometimes you read in the news that people were saved because someone who was in an accident was able to donate lungs, heart, other organs, kidneys… And they were saved” (ID 83468, 68-year-old Filipino American).

## DISCUSSION

The purpose of this study was to examine how Asian Americans exercise individual and relational autonomy in different decisional contexts about organ donation, focusing on *how* participants made their decisions rather than on the decisions themselves. The extant literature has underscored attitudinal and behavioral barriers to organ donation among Asian American populations (25, 41, 43, 44, 55, 56). The low enthusiasm among the current sample of Asian American participants is consistent with prior national study results (41, 57).

### Situational Contexts

The use of situational vignettes and Think Aloud methodology provided insight into the participants’ decision-making frameworks. In the donor registration scenario, more respondents (57.5%) used an individualistic approach. We purport that this is a response to the DMV environment in which most people make this decision where organ donation is essentially distant, unrelated, and potentially unfamiliar occurrence. Nonetheless, a considerable proportion (42.5%) recognized and articulated a need to embed their decision within a framework of shared cultural values and religious beliefs. For one participant (ID 77138), culture dictated that the body must be kept whole and was effectively a barrier for registering as an organ donor, and for another participant (ID 19208), the religious tenets of Jainism was a justification for the opposite decision to register as a donor. These statements referenced a broader set of people (i.e. a family, a religious or ethnic community) and a belief that their decision was ‘answerable’ to others. Unlike when participants exercised individualistic autonomy, these factors were referenced as influences of shared beliefs, social embeddedness, and obligation.

When faced with the hypothetical prospect of donating a family member’s organs, many more respondents were likely to use a relational autonomy framework when making their decision. To illustrate the difference, 42.5% used the relational autonomy framework in the first scenario, but 77.5% did so in the second scenario. Certain aspects are notable. First, the use of individualistic and relational autonomy frameworks is situational for some individuals. Unlike the first scenario in which most respondents had actually made this decision for themselves in a DMV – a usually solitary, official, and impersonal environment – the second scenario prompts respondents to project and decide what to do for someone else, i.e. a close family member. Prior work has pointed to the influence of family in authorizing donation,(25, 44, 45) and the current data confirm that participants actively grapple with reconciling their own individual beliefs about organ donation and the views of their families, formal religious beliefs, and cultural expectations. Respondent 32026 exemplified these considerations. He recognized the emotional weight of the decision and expressed his decision as a function of negotiating the beliefs of family members and those of the deceased. His own beliefs were not articulated. As he and another respondent (ID 71200) indicated, the decision could be further complicated by the relational positions of the involved family members. As the data revealed, autonomy frameworks are dynamic, rather than static and situational. Whereas certain cultural groups may be more likely to use one or the other framework, it is likely that most individuals, no matter what their ethnic or religious affiliations move between these two autonomy frameworks.

### Implications for Practice

This research demonstrates both the limitations of Western notions of autonomy and the need to operationalize an ethical framework that privileges the lived experiences and perspectives of culturally diverse patients rather than of academic philosophical frameworks. Indeed, this study indicates a moderate utilization of individualistic autonomy by study participants across two related but situationally distinct decisional contexts. Furthermore, interviewees acknowledged their decisions as a function of the personal, cultural, and societal elements that constitute their decisional approach. They attributed decisions to the need to align their decisions to others for the sake of family harmony and respect for traditions. Understanding decision-making within the framework of relational autonomy is particularly useful because it accounts for multiple decisional stakeholders without having to necessarily privilege the autonomy of a single decision maker over others. We also demonstrated that, at times, individuals who utilize relational autonomy in one decisional setting will employ individualistic autonomy in another context.

The study’s findings demonstrate the utility of relational autonomy in understanding Asian Americans’ informational and emotional needs when making various decisions about organ donation. While much of the work on relational autonomy has focused on patients at the end-of-life (6, 58, 59), recent research has shown how incorporating relational autonomy into diverse clinical practice settings can aid healthcare providers in assessing patient and family needs and equipping them with sufficient information and support (3, 60–64). Relational autonomy has even been incorporated into Japan’s guideline for Advanced Care Planning (65).

Training organ procurement organization (OPO) staff to recognize the concept of relational autonomy poses potential opportunities. Identifying the use of relational autonomy can inform their interactions with the legal next-of-kin facing the surrogate donation decision by helping them negotiate the decision-making process while remaining respectful of the beliefs and values. Aside from anticipating concerns, such as mistrust in the healthcare system and lower enthusiasm about organ donation among minority groups (44), OPO donation professionals would be prepared to adopt more inclusive approaches involving multiple family members rather than an individual decision-maker. Additionally, a relational approach acknowledges that the needs of a decision maker will fluctuate, and management of donation discussions will require not only regular evaluation of situational needs but also responding with appropriate support. Furthermore, over half of participants in a national study of Asian Americans would authorize donation only if knew the deceased relative’s intentions (41), and the study findings further confirm the importance of knowing a loved one’s donation wishes. Accordingly, public education campaigns in Asian American communities could encourage family conversations about organ donation, designating themselves as donors through their state registries, while also addressing known concerns such concerns about illicit and underground markets for organs (25), keeping the body whole (43, 44, 55, 56), and aversion to discussing topics related to death (25).

### Limitations

This study, of course, has limitations. Because recruitment was facilitated using an online Qualtrics Panel, self-selection bias is possible, as those who participate would necessarily be proficient in and comfortable with computer use, as well as potentially more willing to participate in survey research. The present research sought to confirm insights gleaned from larger and national scale studies using a smaller sample to use qualitative methods that can add richness and context to extant survey data. The role of the demographic characteristics was statistically insignificant, but we recognize that a larger sample could yield different findings.

## CONCLUSIONS

Although this report focuses on an Asian American sample, the concept of the relational autonomy likely has utility in understanding the decision-making process of other populations with regards to organ donation, in other medical settings, and amid emerging phenomena. The current study builds on prior research that suggests the inadequacy of an individualist-driven model of autonomy among Asian and Asian American communities (17, 19–22, 25, 41). Similarly, available literature underscores the role of families in various health settings from many cultural and religious group (66, 67), including those involving organ donation (68–70). Accordingly, future work examining medical decision-making in more broadly may benefit from applying the framework of relational autonomy. For example, the caregiving literature in cancer currently calls for a more family focused perspective although some not specifically situated within a relational autonomy framework (5, 71–73). Relational autonomy maintains that decisions are made adjacent to social, political, and economic forces, rather than in isolation. Thus, the concept of relational autonomy may also be helpful in investigating how patients navigate decisions while healthcare access and delivery change over time.

## Figures and Tables

**Table 1.** Organ Donation Situational Vignettes in Think Aloud Interview

**Scenario 1 - Organ Donor Designation** It is time for you to renew your driver’s license or state ID at your local motor vehicle agency. When you arrive, you are told to complete a form. One of the questions reads, “Would you like to register to be an organ and tissue donor?” What do you decide to do?
**Scenario 2 - Donation at the Bedside** You receive a call from your local hospital and learn that an immediate family member of yours has been rushed to the emergency room after suffering an accident. You go to the hospital immediately. Unfortunately, you are told that your family member will not survive the accident. You are approached about the option to donate your family member’s organs and tissues for transplantation. Your family member did not designate themselves as an organ donor on their driver’s license and does not have a signed organ donor card. You also never discussed the possibility of donation with this family member. What do you do?

**Table 2.** Sample Sociodemographics Information (N=40)

Characteristic	N	(%)
Age (Median 51.5)		
Under 55	21	(52.5)
Sex		
Female	20	(50)
Ethnicity		
Chinese	14	(35.0)
Filipino	11	(27.5)
Indian	10	(25.0)
Korean	3	(7.5)
Pakistani	1	(2.5)
Vietnamese	1	(2.5)
Marital Status		
Married/Cohabitating	25	(62.5)
Never married	12	(30.0)
Widowed	2	(5.0)
Divorced/Separated	1	(2.5)
Nativity		
Not Born/Raised in US	22	(55.0)
Born in US	9	(22.5)
Raised in US	9	(22.5)
Education		
Post-graduate Degree	15	(37.5)
Bachelor’s Degree	17	(42.5)
Some College	5	(12.5)
HS or less	3	(7.5)
Income		
NR/NA	4	(10.0)
Less than $40K	8	(20.0)
$40K - $80K	10	(25.0)
More than $80K	18	(45.0)

**Table 3.** Willingness to Pre-register as an Organ Donor and Decisional Approach

	Pre-register as an organ donor?	
	Yes N (%)	No N (%)
**Individualistic**	14 (53.8)	9 (64.3)
**Relational**	12 (46.2)	5 (35.7)
**TOTAL**	26	14

**Table 4.** Willingness to Authorize Donation and Decisional Approach

	Authorize surrogate donation?		
	Yes N (%)	No N (%)	Undecided N (%)
**Individualistic**	6 (30.0)	1 (7.7)	2 (28.6)
**Relational**	14 (70.0)	12 (92.3)	5 (71.4)
**TOTAL**	20	13	7

## Data Availability

The datasets used and/or analysed during the current study are available from the corresponding author on reasonable request
